# Preparing for effective communications during disasters: lessons from a World Health Organization quality improvement project

**DOI:** 10.1186/1865-1380-7-15

**Published:** 2014-03-19

**Authors:** Laura N Medford-Davis, G Bobby Kapur

**Affiliations:** 1 Section of Emergency Medicine, Baylor College of Medicine, Ben Taub General Hospital Emergency Center, Houston TX, USA

**Keywords:** Disaster communication, Disaster medicine, Disaster planning, Health communication, Health messaging

## Abstract

**Background:**

One hundred ninety-four member nations turn to the World Health Organization (WHO) for guidance and assistance during disasters. Purposes of disaster communication include preventing panic, promoting appropriate health behaviors, coordinating response among stakeholders, advocating for affected populations, and mobilizing resources.

**Methods:**

A quality improvement project was undertaken to gather expert consensus on best practices that could be used to improve WHO protocols for disaster communication. Open-ended surveys of 26 WHO Communications Officers with disaster response experience were conducted. Responses were categorized to determine the common themes of disaster response communication and areas for practice improvement.

**Results:**

Disasters where the participants had experience included 29 outbreaks of 13 different diseases in 16 countries, 18 natural disasters of 6 different types in 15 countries, 2 technical disasters in 2 countries, and ten conflicts in 10 countries.

**Conclusion:**

Recommendations to build communications capacity prior to a disaster include pre-writing public service announcements in multiple languages on questions that frequently arise during disasters; maintaining a database of statistics for different regions and types of disaster; maintaining lists of the locally trusted sources of information for frequently affected countries and regions; maintaining email listservs of employees, international media outlet contacts, and government and non-governmental organization contacts that can be used to rapidly disseminate information; developing a global network with 24-h cross-coverage by participants from each time zone; and creating a central electronic sharepoint where all of these materials can be accessed by communications officers around the globe.

## Background

During and after a disaster, effective communications must coordinate response efforts in order to limit secondary morbidity and disease [1]. Organizations must communicate early and frequently with multiple stakeholders to prevent panic and implement an orderly response plan [2]. The government and other decision makers need to know what response efforts are ongoing, and what type of further assistance is required where in order to coordinate relief. Health professionals want to know which health risks or diseases are increased in the current environment, how best to advise their patients, and how they can stay informed of emerging disease trends while working in the field. The public wants to know how to obtain assistance, what ongoing personal risks they face, and how they can protect themselves and their families [3]. Platforms for this type of health messaging include press releases and media interviews, Internet articles and social media, town hall forums, and frequent timely communication among responders.

Each disaster serves as a learning opportunity for how to communicate better in the next disaster. Several retrospective studies have tried to document these lessons by determining how the public understood the messages that were communicated to them during a recent disaster [4-11]. Gaps in a disaster communication plan such as technical or complex instructions [4] can leave groups vulnerable to misunderstanding the message, while methods of dissemination [5-8] and demographics [7,9] can result in the message never reaching certain target populations. Other studies have focused on learning lessons from the groups responding to the disaster, including healthcare professionals [8,10] and US governmental agencies [11].

A large body of risk communications literature has gone beyond the piecemeal focus on each individual disaster to educate on overarching best methods of health messaging [12,13]. However disaster communications has been criticized because communications preparedness remains underdeveloped [14]. An expert Delphi study published in 2012 came to the consensus that despite all the existing literature, there is still a lack of understanding about communication, identifying communication as a top three priority area for further disaster management research [15].

## Methods

The primary author interviewed WHO Communications Officers who had responded to prior disasters using an open-ended survey. The primary author was employed by WHO and based in Switzerland at the time of survey administration and data review. This quality improvement project represents a non-sensitive survey approved by the WHO Communications Department Head, which was used for internal evaluation and improvement of existing WHO procedures, and as such was subject to the regulations of WHO, which does not have an IRB for internal projects.

A communications officer at WHO is responsible for developing and publishing communication and advocacy material for the organization, serving as a spokesperson for the organization, developing and implementing a strategic corporate communication plan, and supporting member countries to develop and implement communications. The communications officers work for WHO offices primarily leading daily communications activities (e.g., World Health Day campaign, release of new WHO guidelines, etc.), but in the case of a disaster may be deployed to support disaster communications in the affected area.

A senior WHO employee with extensive communications and disaster experience at WHO contributed an initial list of 15 potential participants. Participants were then asked to refer other participants with disaster experience using snowball sampling methodology. A total of 31 potential participants were identified and contacted via corporate email to request their participation. Twenty-eight people responded; 2 were excluded due to scheduling conflicts and 26 were formally interviewed by phone, by video conference, or in person.

In-depth interviews were conducted in English. Participants were asked a series of closed- and open-ended questions from a structured survey template about their experiences responding to disasters as communications officers on behalf of WHO (Additional file 1). Interviews lasted approximately 75-90 min. All of the interviews were digitally audio-recorded with the participants’ permission. Data were entered into a Microsoft Excel spreadsheet, version 14.0.6129.5000 (Microsoft Corp., Redmond, WA, USA).

Analysis included: (1) descriptive statistical analysis to objectively characterize the incidence of different types of disaster experience; (2) a careful re-listening to the interview recording to fully understand participant’s perspectives; (3) coding of the responses to each open-ended question into different thematic categories based on word repetitions and key words in context [16]; (4) extraction of data using pawing [16] to determine the most frequently reported barriers and augmenters of effective disaster communication; (5) extraction of data using pawing to determine the most commonly reported themes of disaster communications that represent transferable knowledge between different disasters. Some participants gave responses that did not fit into the key word themes of any other participants; these were recorded as new themes at a frequency of one to exhaust all possible themes. Themes were ranked in importance based on their frequency of appearance in the responses, with the addition of one single-frequency recommendation theme determined to be important based on the researchers’ prior literature review of the topic and knowledge of the structure of the WHO. Results were used by the WHO Communications Department to develop new protocols for future WHO disaster response communications.

## Results

Twenty-six WHO Communication Officers from Headquarters (HQ) in Geneva, all 6 regional offices (RO), and 11 country offices (CO) participated. The majority (*N* = 22; 85%) had experience in multiple disasters. Participants had responded to both acute and chronic disasters, defined as whether the disaster had happened less or more than 3 months prior to deployment. Most communications officers were deployed close to the disaster epicenter: to the field, to the nearest country office, or to a combination of both. Half were deployed within 3 days to 1 month after the disaster began, but just three arrived within the first 72 h after the disaster (Table 1). The disasters where they had worked included 29 disease outbreaks of 13 different diseases in 5 regions and 16 countries, 18 natural disasters of 6 different types (e.g., tsunami, earthquake, flood, etc.) in 5 regions and 15 countries, 2 technical disasters in 2 regions and 2 countries, and 10 conflicts in 3 regions and 10 countries.

**Table 1:** Crisis types

**Characterization**	***N *** **(Percent)**
Crisis length	
Acute	21 (81%)
Chronic	5 (19%)
Crisis type	
Natural disaster	13 (50%)
Outbreak	11 (42%)
Conflict	2 (8%)
Deployment location	
Field	10 (39%)
Country office	7 (27%)
Field and country office	5 (19%)
Regional office	3 (11%)
Headquarters	1 (4%)
Arrived how long after crisis started	
First 72 h	3 (12%)
3 days–1 week	6 (23%)
1 week–1 month	6 (23%)
Greater than 1 month	11 (42%)

Qualitative responses from the communications officers resulted in a list of core communications priorities for WHO during a disaster response (Table 2), recommendations for hiring a competent communications officer who can be deployed for disaster response (Table 3), and recommendations for trainings a communications officer should receive prior to deployment (Table 4). In addition, the communications officers provided recommendations for methods to build communications capacity that could be undertaken prior to a disaster to improve preparedness (Table 5).

**Table 2:** Suggested communications role for WHO during a disaster response

**Communications role**	***N *** **(percent)**
**Disseminate information and products of communication**	**26 (100%)**
Develop and maintain internal and external contact lists	3 (11.5%)
Take meeting minutes; share information between WHO team members	13 (50%)
Send daily updates to RO and HQ	17 (65%)
Write situation reports	19 (73%)
Develop a communications strategy	24 (92%)
**Media liaison**	**26 (100%)**
Draft talking points and Q&As	17 (65%)
Draft press releases	14 (54%)
Organize press conferences	13 (50%)
Respond to media queries	10 (38%)
Media monitoring	5 (19%)
**Advocacy and resource mobilization**	**17 (65%)**
Document the crisis response	13 (50%)
Develop feature stories	11 (42%)
Update the website	9 (35%)
Write donor reports	3 (11.5%)
Write grant proposals	1 (4%)
**Liaison to Ministry of Health**	**13 (50%)**
Provide technical support for government communications; give public visibility to government response efforts	7 (27%)
Coordinate data and communications strategy	6 (23%)
Health promotion and social mobilization	12 (46%)

**Table 3:** Recommended skillset for a disaster communications expert

**Recommendations**	***N *** **(percent)**
Professional skills	
Managerial skills: able to lead a communications team to coordinate a communications strategy	18 (70%)
Effective writing and editing skills	15 (58%)
Media relations experience	12 (46%)
Analytical skills: able to synthesize large volumes of technical information into concise common language	3 (12%)
Technical skills	
Photography and videography: able to record and edit	24 (92%)
Public health literacy	12 (46%)
Computer proficiency	6 (23%)
Working use of English and preferably the local language	7 (27%)
Interpersonal skills and helpful personality traits	
Team player: able to work on a team with people from diverse cultural and professional backgrounds	14 (54%)
Diplomatic and respectful in complex socio-cultural-political contexts	12 (46%)
Remains calm under stress	10 (38%)
Willing to work in hardship conditions	10 (38%)
Flexible	8 (31%)
Proactive	6 (23%)
Able to multi-task	6 (23%)
Resourceful, able to improvise	4 (15%)
Able to quickly assess a rapidly evolving situation and act quickly and decisively	4 (15%)

**Table 4:** Trainings for a communications officer prior to deployment

**Recommendations**	***N *** **(Percent)**
Communications	12 (46%)
Designing a crisis communications strategy	
How to manage and coordinate an emergency communications team	
Presentation and media spokesperson skills	7 (27%)
Humanizing statistics for a general audience	
Simulation in a rapidly evolving, high-stress environment	
Disaster standards and guidelines	7 (27%)
Sphere Handbook	
OSHA	
UN and health cluster structure	
Public health	8 (31%)
Epidemiology, basic statistics	
Types of disasters and their health consequences	
Myths and realities of health during a disaster	
Personal effectiveness	6 (23%)
Basic life support and first aid	
Security precautions	
Coping with stress	
IT	3 (12%)
How to use a satellite phone	
Establishing phone and internet connections in remote locations	

**Table 5:** Recommendations to improve communications capacity prior to a crisis

**Recommendation**	***N *** **(percent)**
Develop and pre-write or pre-record common public service announcements in multiple languages on questions that frequently arise during crises	7 (26%)
Maintain a database of statistics for various regions and types of crisis	2 (8%)
Maintain lists for all frequently affected countries and regions of the locally trusted media outlets and sources of information	3 (12%)
Maintain email listservs that can be used to rapidly disseminate information to your organization’s employees, international media outlet contacts, government Ministries of Health, and non-governmental organization contacts	3 (12%)
Develop a global network with 24-h cross-coverage by participants from each time zone	2 (8%)
Create a central electronic sharepoint where all of these materials can be accessed by communications officers around the globe when crisis strikes	1 (4%)

## Discussion

One hundred ninety-four member states turn to WHO for guidance and assistance in response to disasters that include not only disease outbreaks, but also natural disasters, man-made disasters, and conflicts [17]. Post-disaster communication is one area of expertise for which WHO provides support to member countries. WHO also convenes international experts to reach public consensus on priority topics, and it recently convened an expert panel on the topic of communications during disease outbreaks [18].

**Figure 1:**
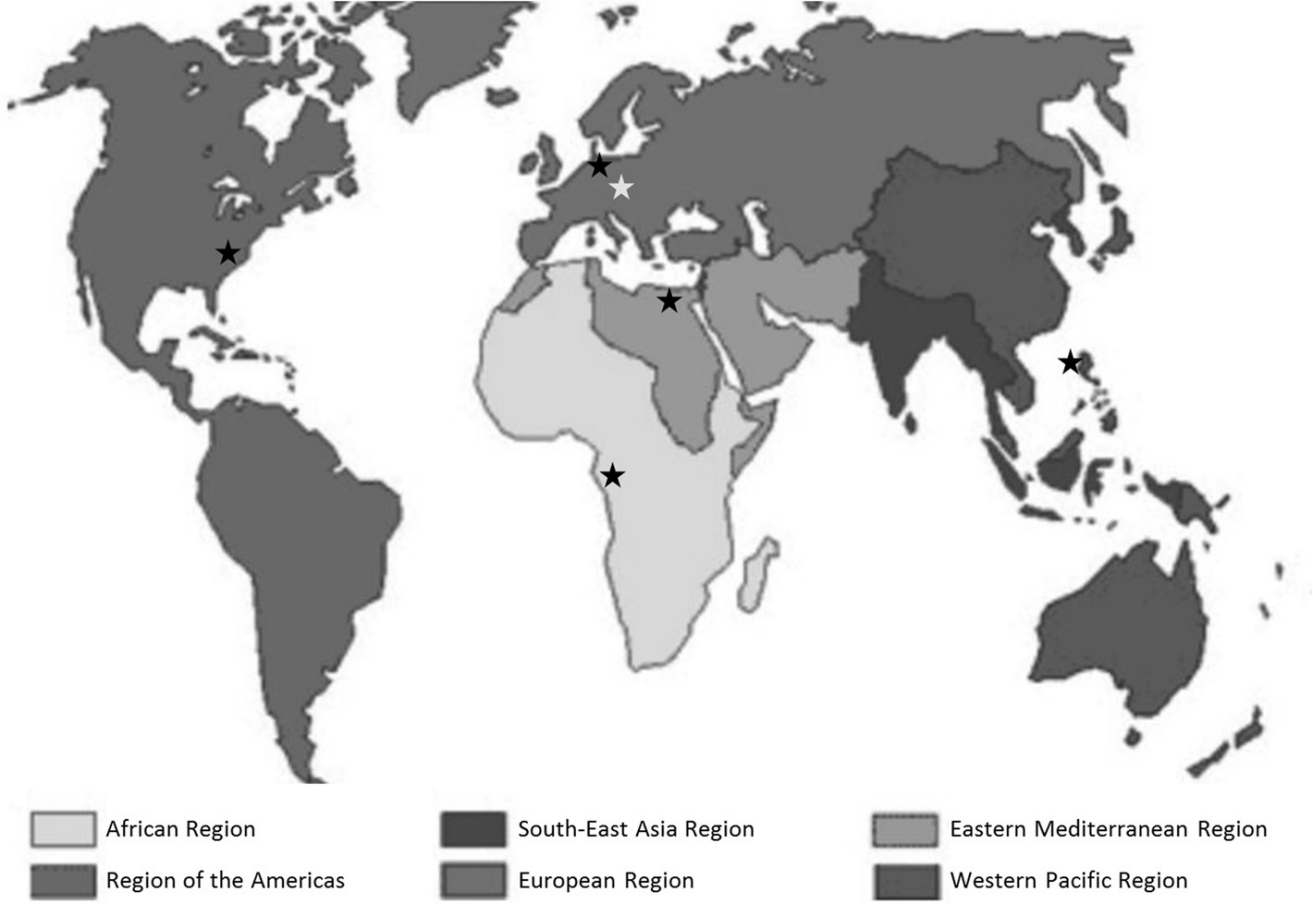
**Location of WHO regions and headquarters **[20]**.**

WHO Headquarters is subdivided into clusters that operate relatively independently [19]. Different clusters respond to different types of disasters and sometimes even to different aspects of the same disaster. For example, the Humanitarian Action in Crisis (HAC) cluster responds to natural disasters and conflicts, while the Global Alert and Response Network (GAR) responds to disease outbreaks. After the earthquake in Haiti in 2010, HAC initially responded, but GAR joined relief efforts later that year when a cholera outbreak began. In addition, WHO consists of six regions each coordinated from a RO (Figure 1) [20]. At the regional level there are also clusters, although their specific titles and functions vary from region to region. Finally, COs have large variations in the number and specialized scope of staff. Countries with larger WHO operations have larger offices, while some countries do not house an in-country office at all.

A small number of communications officers working at different levels (country, regional, or headquarters) and in the different clusters are consistently deployed throughout this global network to respond to disasters. Usually the CO where the disaster occurred requests additional support from their RO or HQ if they do not have their own communications officer or if there is too much work for one officer. Each individual officer possesses vast experiential knowledge about disaster response and communications during disasters in particular. This study was organized by the Department of Communications (DCO) cluster, which leads communications at HQ. Its objective was to gather and utilize this experiential knowledge to formulate best practices for communication during a disaster that could be used to develop WHO policies and procedures, as well as to develop a training program to improve preparedness for deployment of WHO Communications Officers to disasters.

WHO’s main role during a disaster is to support the local government’s Ministry of Health (MoH) and to co-chair the United Nations Health Cluster along with the local MoH. In this role, WHO communications officers are responsible for sharing information about the response efforts among the different member organizations of the Health Cluster. They create and update a detailed situation report and distribute it frequently to all cluster members. The report maps which organizations are providing which health services to each affected area, where gaps still remain in services, and the joint action plan to fill those gaps. For this role, it is helpful to have the contacts at each major health cluster organization included in electronic listservs so that a communications leader can rapidly send an internal message to government partners at the Ministry of Health and to non-governmental organization partners who are participating in the response effort.

Where possible and depending on the size of an organization, additional team members can be assigned to support the communications activities of those in the field through a global network with 24-h cross-coverage from offices in other time zones. While those working in the field sleep, communications officers at a headquarters in another time zone where it is still daylight can respond to international media queries on their behalf. Current WHO practice is to hold daily teleconferences between the communications officer in the field or CO and the other communications officers supporting them from the RO and/or HQ to summarize the situation and assign the work that needs to be completed. Additional communications occur as needed throughout the day via email and/or telephone. Raw photo or video footage from the field can be emailed to the office-based staff for editing prior to further distribution.

Perhaps the most interesting and broadly applicable finding of this study is the recommendation for how to prepare disaster communications ahead of time, which included several useful and novel methods that can be adapted by any organization. Respondents noted that similar questions arose in seemingly different disasters and suggested preparing the answers to the most common problems in advance. One example is allaying the public’s fear of dead bodies spreading disease, since several experts pointed out that bodies do not pose an imminent risk when killed by a natural disaster or conflict rather than an infectious disease. Another example is a message to the international public to refrain from sending bulk donations of used goods and instead to wait for a request of which specific goods are needed and where. Additional examples include warnings about generator use to prevent carbon monoxide poisoning, and messages about safe handling of waste and how to sanitize water when plumbing has been compromised. Such public health messages could be written and recorded for television, radio, and social media in multiple languages ahead of time (Table 6).

**Table 6:** Examples of disaster messages to prepare in advance

	**Storms (hurricane, tornado)**	**Floods, (Tsunamis)**	**Conflicts**	**Disease outbreak**	**Chemical disaster**
Dead bodies killed by natural disaster and conflict do not carry disease	X	X	X		
Avoiding waterborne illness; sterilizing water	X	X		X	X
Basic wound care and first aid	X	X	X		
Vaccines and immunizations	X	X	X	X	
Personal protective equipment to avoid secondary injury	X	X		X	X
Power outage safety: staying warm (or cool depending on the local weather conditions)	X	X	X		
Power outage safety: carbon monoxide poisoning	X	X	X		
Safety around loose power lines	X	X			
Identifying and avoiding gas leaks	X	X			
Do not send bulk donations; wait for specific requests	X	X	X		

Developing a databank of basic statistics about the local population, health status, and disaster risk for areas most frequently affected by disaster was also suggested. Pairing each statistic with a brief descriptor written in human factor terminology makes it ready for immediate use in communications. As an example of human factor terminology, in an area commonly afflicted by flooding, rather than stating that the incidence of floods is 21% per lifetime, it is better to state that one of every five persons will be affected by a flood in their lifetime.

For those regions frequently affected by particular disaster types, media surveillance should be undertaken regularly as to which sources of information the local public trusts. These sources may include a particular television network, radio station, or even a community leader or religious authority. Text messaging and/or social media can also augment the number of people reached. During a disaster, these trusted sources are the outlets that should be targeted when disseminating health messages to the public to ensure that no group misses the message. Listservs of these local media and community contacts should be created and maintained in advance of any disaster for quick dissemination of press releases and public health messages.

Another recommendation to improve future coordination within WHO was to store all of the documents mentioned above including common messages, statistics, and listservs with important contacts in a central electronic repository utilizing “cloud technology” such as Sharepoint, Google Docs, or Dropbox where they can be readily accessed and updated by all of the organization’s officers working from around the globe. Other areas which were identified for improvement that are more specific to internal WHO processes included the lack of a defined on-call system for deployment and the lack of a formal briefing or debriefing process. The need to develop more formal training to expand the list of experts qualified for deployment led to a list of suggested trainings that would help prepare a communications officer for first-time deployment (Table 4).

### Limitations

This is a retrospective study composed of a convenience snowball sample rather than a randomized sample of all possible participants. Because people were asked very open-ended questions without prompting, the few responses that were offered by nearly 100% of respondents likely indicate a very strong relevance, but it is difficult to draw scientific conclusions about responses given by <50% of respondents since other respondents might have agreed had they been specifically questioned on the value of these ideas. This is a preliminary data-gathering project that presents interesting ideas that should be further evaluated in a more rigorous study, ideally convening disaster communications experts from a variety of organizations.

## Conclusions

Many communications tasks can and should be undertaken prior to a disaster to improve preparedness. Some of these tasks represent common sense, while others may be more novel. Investing time and manpower now to improve an organization’s communications capacity can save time in disseminating key messages to minimize chaos and coordinate stakeholders once disaster strikes.

## Abbreviations

WHO: World Health Organization; HAC: Humanitarian action in crisis; GAR: Global alert and response network.

## Competing interests

The authors declare that they have no competing interests.

## Authors’ contributions

LMD performed the surveys and theme-generating data analysis. GBK contributed to the project methodology and design. LMD and GBK wrote the manuscript. All authors read and approved the final manuscript.

## Authors’ information

LMD is Chief Resident at the BCM Emergency Medicine residency program and has been selected as a Robert Wood Johnson Fellow for 2014.

GBK is the founding emergency medicine residency program director at BCM and is the founding director of the Center for Globalization at BCM.

## Supplementary Material

Additional file 1 Data collection instrument: interview questions. Click here for file
