# Supplementary material for: Preparing for effective communications during disasters: lessons from a World Health Organization quality improvement project
Source: Int J Emerg Med. 2014 Mar 19;7:15. doi: 10.1186/1865-1380-7-15 (PMC4000058; doi:10.1186/1865-1380-7-15)
Supplement: Additional file 1 — Data collection instrument: interview questions. [file 1865-1380-7-15-S1.docx]

**Additional file 1: Data Collection Instrument: Interview Questions**

You have experience in which crises?

Acute or chronic?

Conflict, natural disaster, or disease outbreak?

At which level did you work? (Field, Country office, Regional office, Headquarters, or with the UN?)

How long after the crisis onset were you deployed?

How much notice did you have before deployment?

What was the total length of time you were deployed?

Were you briefed, and if so, by whom and on what topics?

After assignment before departure?

Upon arrival?

After deployment ended?

Did you receive psychosocial debriefing after deployment?

Did you print or read anything to brief yourself on the situation before deployment?

Describe any field support.

Describe your housing situation.

How was your performance during deployment assessed?

How is a communications officer selected for deployment?

What were WHO’s priorities during the crisis?

How did the UN Health Cluster operate during the crisis?

Who was the leader?

How often did the cluster meet?

Who attended cluster meetings?

Besides the Health Cluster, what other meetings were ongoing in the field?

What did the communications officer do during the crisis?

What are Core communications tasks?

What other tasks were communications officers asked to do?

Who approved the communications work products?

Did all products need approval from a higher level?

Who did the communications officer report to?

How is work shared between the different levels of the WHO?

Did the communications officer work more with the UN and Health Cluster or with WHO?

How does the communications officer fit into the field team hierarchy?

How many communications officers were on your team? If >1, how did you distribute work between communications officers?

If you were writing the Terms of Reference to hire a communications officer for deployment, what are the core competencies and skills you would include?

If you were talking to a communications officer being deployed tomorrow, what tips would you give them?

What should you pack?

What trainings should a communications officer have prior to deployment?

What recommendations do you have to improve the communications and deployment system?

Who else would you recommend I speak with to inform this project?
